# Metabolic Reprogramming of Phospholipid Fatty Acids as a Signature of Lung Cancer Type

**DOI:** 10.3390/cancers16193320

**Published:** 2024-09-28

**Authors:** Marija Paunovic, Ana Stojanovic, Biljana Pokimica, Jasmina Debeljak Martacic, Zorica Cvetkovic, Nebojsa Ivanovic, Vesna Vucic

**Affiliations:** 1 Centre of Research Excellence in Nutrition and Metabolism, Institute for Medical Research, National Institute of Republic of Serbia, University of Belgrade, 11000 Belgrade, Serbia; biljana.pokimica@imi.bg.ac.rs (B.P.); jasmina.martacic@imi.bg.ac.rs (J.D.M.); 2 Department of Pulmonology, University Hospital Medical Center (UHMC) “Bezanijska kosa”, 11000 Belgrade, Serbia; dr.anastojanovic@yahoo.com; 3 Faculty of Medicine, University of Belgrade, 11000 Belgrade, Serbia; zcvetkovic06@gmail.com (Z.C.); ivanovicnebojsadr@gmail.com (N.I.); 4 Department of Hematology, University Hospital Medical Center Zemun, 11080 Belgrade, Serbia; 5 Department of Surgical Oncology, University Hospital Medical Center (UHMC) “Bezanijska kosa”, 11000 Belgrade, Serbia

**Keywords:** non-small cell lung cancer, small cell lung cancer, fatty acids profile, tumor tissue, phospholipids

## Abstract

**Simple Summary:**

Lung cancer is one of the leading causes of cancer-related mortality. Non-small cell lung cancer (NSCLC) and small cell lung cancer (SCLC) differ in aggressiveness, proliferation speed, metastasis propensity, and prognosis. Since tumor cells notably change lipid metabolism, especially phospholipids and fatty acids (FA), this study aimed to identify FA alterations in lung cancer tissues. We detected significant changes in lipid metabolism in both lung cancer types compared to healthy tissues (especially higher levels of pro-inflammatory arachidonic acid), and we pointed out differences in FA profiles in tissue between SCLC and NSCLC. Our findings can be useful for further research concerning molecular lung cancer therapies.

**Abstract:**

**Background:** Lung cancer is one of the leading causes of cancer-related mortality. Non-small cell lung cancer (NSCLC) and small cell lung cancer (SCLC) differ in aggressiveness, proliferation speed, metastasis propensity, and prognosis. Since tumor cells notably change lipid metabolism, especially phospholipids and fatty acids (FA), this study aimed to identify FA alterations in lung cancer tissues. **Methods**: Our study included patients with newly diagnosed, histologically confirmed SCLC (*n* = 27) and NSCLC (*n* = 37). Samples were collected from both malignant and healthy tissues from each patient, providing they were within subject design. **Results**: In both NSCLC and SCLC tumor tissues, FA contents were shifted toward pro-inflammatory profiles, with increased levels of some individual n-6 polyunsaturated FA (PUFA), particularly arachidonic acid, and elevated activity of Δ6 desaturase. Compared to healthy counterparts, lower levels of alpha-linolenic acid (18:3n-3) and total saturated FA (SFA) were found in NSCLC, while decreased levels of linoleic acid (18:2n-6) and all individual n-3 FA were found in SCLC tissue in comparison to the healthy tissue control. When mutually compared, SCLC tissue had higher levels of total SFA, especially stearic acid, while higher levels of linoleic acid, total PUFA, and n-3 and n-6 PUFA were detected in NSCLC. Estimated activities of Δ6 desaturase and elongase were higher in SCLC than in NSCLC. **Conclusions**: Our findings indicate a notable impairment of lipid metabolism in two types of lung cancer tissues. These type-specific alterations may be associated with differences in their progression and also point out different therapeutic targets.

## 1. Introduction

Lung cancer is one of the leading causes of cancer-induced death, causing 1.8 million deaths per year. It is the most prevalent cancer in both men and women, diagnosed in 12.4% of all cancer cases [1], and, as such, represents a great public health burden. Non-small cell lung cancer (NSCLC) is the most common lung cancer, contributing to 75–85% of total cases, and is most often diagnosed in advanced stages [2]. According to the World Health Organization (WHO), NSCLC is classified into three main categories, including squamous cell carcinoma, adenocarcinoma, and large cell carcinoma, and this heterogeneity makes it difficult to establish pathology, diagnosis, and therapeutic decision [3]. Conversely, small cell lung cancer (SCLC) accounts for about 15% of all lung cancers and is characterized by an exceptionally high proliferative rate, a strong tendency for early metastasis, and poor prognosis [4]. When comparing these two types of lung cancers, SCLC grows and spreads faster than NSCLC and is less likely to be cured even with novel treatment strategies [5].

Phospholipids (PL), forming the cell membrane bilayer, are vital for cell membrane structure and signaling. The properties of PL depend on their fatty acids (FA) composition [6]. One of the characteristics of malignant cell growth is metabolic reprogramming, including altered lipid metabolism, which influences cancer cells’ survival, migration, and proliferation. Changes in membrane PL FA composition can affect tumor behavior, including cancer growth, progression, and survival. The activity of enzymes responsible for lipogenesis, such as ATP citrate lyase (ACLY) and fatty acid synthase (FAS), is enhanced in tumor cells [7]. The upregulation of FAS activity is involved in most of the cancer progressions. Moreover, it is elevated in a stage-dependent manner and is associated with a worsened prognosis [8]. Furthermore, it is well documented that carcinogenesis is often accompanied by the increased conversion of saturated FA (SFA) to monounsaturated fatty acids (MUFA) by stearoyl-CoA desaturase-1 (SCD1). This leads to a significantly higher MUFA/SFA ratio in the malignant area than in the adjacent normal area for several cancers as a means to mitigate the intrinsic cytotoxicity of SFA and increase metastatic properties by enriching membrane fluidity [9]. Polyunsaturated FA (PUFA) also play an important role in both the induction and prevention of carcinogenic processes. It is known that n-3 PUFA, as precursors of anti-inflammatory mediators, have antitumor effects, while n-6 PUFA act in the opposite direction since their products are pro-inflammatory eicosanoids. High n-6 levels in blood and tissue lead to inflammatory processes that could trigger cancer development [10]. Consequently, altered FA distribution is commonly found in the plasma and red blood cell membranes of cancer patients [11,12,13]. This observed trend was also reflected in the plasma of lung cancer patients when compared to the healthy controls, as well as in differences between early and advanced stages of carcinoma [14]. Additionally, significant changes in blood lipid profile were noted in the same patients during different phases of therapy [15]. On the other hand, data on lipid changes in tumor tissue are very limited, and since the samples can only be obtained through a biopsy, which provides a small and often insufficient amount of tissue, many patients are not eligible for biopsies, and this procedure can lead to complications [16].

More comprehensive data on the distribution of PL within the tumor cells would be crucial for understanding the influence of lipids and their metabolism in carcinogenesis, disease progression, and prognosis. Furthermore, the enzymes involved in their synthesis and regulation of their activity might be potential therapeutic targets for new cancer therapy. Therefore, the aim of this study was to compare FA profiles in phospholipids between healthy and tumor tissues in NSCLC and SCLC subjects, as well as to compare these two different types of lung cancer tissues with each other. Furthermore, one of the goals was to compare whether the differences in distribution of PL in these two tumor tissues might be related to different clinical prognoses.

## 2. Materials and Methods

### 2.1. Study Population

This study involved 64 patients (44 men and 20 women) with newly diagnosed treatment-naïve NSCLC (*n* = 37) or SCLC (*n* = 27), who were recruited at the Clinical Center of Serbia “Bežanijska Kosa”. The majority of the patients had an advanced stage of the disease; only 2 patients with NSCLC had II stage, while 14 of them had III and 21 patients had IV stage (therefore, 35 out of 37 patients had III and IV stage). Furthermore, about 80% of SCLC patients (22 out of 27) had an extensive stage of the disease. In the SCLC group, there were 6 female patients (22.2%), and in the NSCLC group, there were 14 (37.8%).

The average age of participants was 63 years (45–80), and 86% of them had a smoking history. Excluding criteria were: the presence of other cancer types, metastasis, occurrence of liver, kidney, and other serious chronic non-malignant diseases, survived heart attack in the last 6 months, consumption of drugs that influence lipid metabolism, taking fish oil or evening primrose (*Oenothera biennis*) supplements, and poor performance status (ECOG score less than 1). Blood samples were collected after overnight fasting. Lipid status, C reactive protein (CRP), and fibrinogen concentration were analyzed from the serum using a Clinical Chemistry Analyzer (Cobas c111, Roche Diagnostics, Basel, Switzerland) and Roche Diagnostics kits, following the manufacturer’s instructions.

### 2.2. Ethical Statement

This study was approved by the Ethical Review Board of the Clinical Hospital Center Zemun in accordance with the principles of the Declaration of Helsinki (No. 145/1, Date 21 April 2016). All participants signed informed consent.

### 2.3. Tissue Collection

All patients, following the assessment of their general condition, hemodynamic status, laboratory parameters, and coagulation status, underwent invasive pulmonary diagnostics. Based on a chest X-ray and multi-slice chest CT scan, an appropriate diagnostic procedure was determined. Patients received local anesthesia, followed by flexible bronchoscopy using an Olympus BF type TE2 flexible bronchoscope (Olympus Medical Corporation, Tokyo, Japan), (working channel width 2 mm). Small forceps were used to take a histopathological sample from the site of healthy bronchial mucosa and then from the site of tumor changes. Biopsied tissue from the unaffected site and from the site of tumor changes was frozen at −60 °C and stored for further analysis. All patients in the study served as their own controls, adhering to the principles of self-comparison within the research framework.

### 2.4. Tissue Phospholipid Fatty Acids Extraction

Total lipids from tissues were isolated by using a mixture of solvents and chloroform-methanol 2:1 (*v*/*v*) with added butylated hydroxytoluene (BHT) to prevent lipid oxidation. Tissue extracts were prepared by using 0.5 g of tissue homogenized in a chloroform-methanol solution and left to extract for 3 h in the refrigerator. Into 100 μL of homogenate was added 4.5 mL of solvent, followed by the passage of the resultant extract through sodium sulfate and subsequent evaporation to dryness. The dry extract was dissolved in 0.2 mL of a chloroform-methanol mixture and used for the chromatographic separation of lipid classes. Lipid classes were separated by one-dimensional thin-layer chromatography (TLC) on silica gel (GH600, Merck, Hoddesdon, UK) with a thickness of 0.5 mm. The total lipid extract from tissue was applied to a pre-activated plate. For the mobile phase, a mixture of petroleumether-diethylether-acetic acid (87:12:1, *v*/*v*/*v*) was used. Phospholipid fractions were separated from the plate, extracted with hexane, and used for further esterification.

### 2.5. Phospholipid Composition

Phospholipid fractions were extracted by direct transesterification with 3 M HCl in methanol using the previously explained protocol [17]. The obtained methyl esters were extracted with hexane, evaporated to dryness, and further analyzed by gas chromatography (GC). PL composition was determined using a Shimadzu 2014 gas chromatograph with a flame ionization detector (Shimadzu 2014, Waltham, MA, USA). The PL were separated on a Restek Rtx 2330 capillary column (Restek, Bellefonte, PA, USA), with a 60 m × 0.25 mm inner diameter and a film thickness of 0.2 μm, and identified through comparison with the retention time of commercial standards (PUFA-2 and/or 37 FAME mix, Supelco, Bellefonte, PA, USA). The PL composition was articulated as a percentage of the total FA identified. The activities of desaturases and elongases were estimated as the product/precursor ratio of FA as follows: 16:1n-7/16:0 for SCD1, 18:1n-9/18:0 for SCD2, 20:4n-6/20:3n-6 for Δ5 desaturase, 20:3n-6/18:2n-6 for Δ6 desaturase, and 18:0 to 16:0 ratio for elongase activity [18].

### 2.6. Statistical Analysis

Statistical analysis was performed using SPSS 23.0 software (SPSS Inc., Chicago, IL, USA). The normality of the variables was assessed using the Shapiro-Wilk test. Comparisons of means between groups were conducted using either the *t*-test or the Mann-Whitney U test, depending on the distribution of the variables. For comparisons between healthy and malignant tissues, the paired t-test was applied for normally distributed variables, while the Wilcoxon test was used for variables with an asymmetric distribution. Values are presented as mean ± standard deviation (SD). The level of significance was set at *p* ≤ 0.05.

## 3. Results

The obtained results show that patients with SCLC had statistically significantly higher triglyceride levels (*p* < 0.01) than NSCLC patients, while the levels of other examined serum lipids and inflammation parameters were not found to differ, as shown in Table 1.

The FA composition of PL in NSCLC tissue revealed significant differences when compared to the healthy lung tissue of the same patients (Table 2). A higher proportion of the following FA: 18:1n-7, 20:3n-6 (DGLA), 20:4n-6 (AA), 22:4n-6 (DTA), PUFA, and n-6 PUFA, was found in malignant tissue. The FA 16:0, 18:3n-3 (ALA), and SFA were significantly less represented in NSCLC tissue compared to healthy tissue.

Through a comparison of the estimated desaturase and elongase activities in healthy and malignant tissue, a statistically significant elevation in desaturase Δ6 activity within malignant tissue relative to the healthy lung tissue of the same patients afflicted with NSCLC was identified, as shown in Table 3.

The FA composition of PL in both the healthy and malignant tissue of each individual subject with SCLC significantly diverged, as shown in Table 4. Elevated levels of the following FA were observed in the tumor tissue: 18:1n-9, 18:1n-7, 20:4n-6, 22:4n-6, and MUFA, as well as the ratio of n-6/n-3 PUFA. Conversely, PL in SCLC malignant tissue exherted a reduced proportion of 18:2n-6 (LA), 20:5n-3 (EPA) (lower but not significant), 22:5n-3 (DPA), 22:6n-3 (DHA), total n-3 PUFA, and EPA/AA ratio.

We also compared the activities of desaturases and elongases in healthy and malignant tissue in patients with SCLC and observed that desaturases Δ5, Δ6, and Δ9 activities were elevated in tumor tissue in comparison to the control (Table 5).

**Figure 1:**
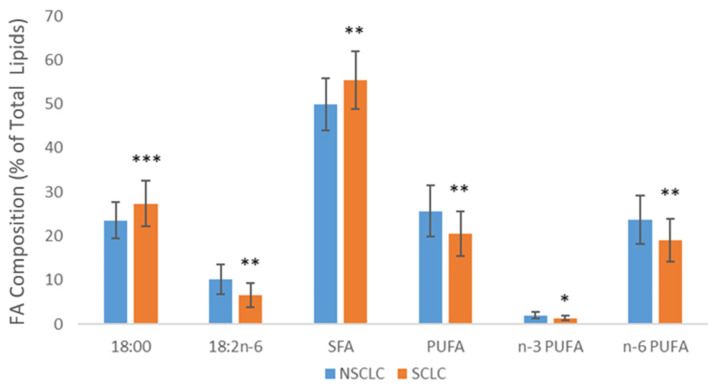
Differences in the levels of fatty acids in SCLC and NSCLC tumor tissue. SFA—saturated fatty acids; PUFA—polyunsaturated fatty acids; NSCLC—non-small cell lung carcinoma; SCLC—small cell lung carcinoma; * *p* < 0.05, ** *p* < 0.01, *** *p* < 0.001.

**Figure 2:**
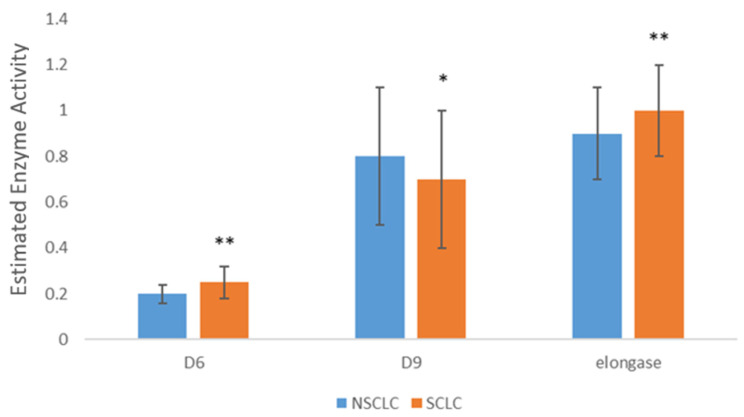
Differences in desaturases and elongase activity SCLC and NSCLC tumor tissue. SFA—saturated fatty acids; PUFA—polyunsaturated fatty acids; NSCLC—non-small cell lung carcinoma; SCLC—small cell lung carcinoma; D6—Delta 6 desaturase; D9—Delta 9 desaturase; * *p* < 0.05, ** *p* < 0.01.

Further comparison of FA composition in SCLC and NSCLC tissues revealed significant differences between these two types of lung carcinomas. Figure 1 displays statistically significant differences in the levels of some individual FA and their categories. Higher levels of stearic acid and SFA were found in SCLC tissue, while higher levels of LA, PUFA, n-3 PUFA, and n-6 PUFA were detected in NSCLC tissue. The estimated activities of Δ6 desaturase and elongase were higher in SCLC tissue, while the activity of Δ9 desaturase was higher in NSCLC tissue (Figure 2). Fatty acids and the estimated activities of enzymes that showed no differences between SCLC and NSCLC are not presented.

## 4. Discussion

In this study, we analyzed the FA profile in the PL of malignant and healthy tissues of lung cancer patients and compared FA composition in the two most common lung cancer types—NSCLC and SCLC. In non-malignant cells, de novo FA synthesis occurs rarely, and cells preferentially use exogenous FA in order to satisfy their metabolic needs [19]. Cancer cells divide considerably faster than healthy cells and therefore have higher needs for energy and macronutrients. To meet the increased requirements, malignant cells undergo major metabolic modifications, and it is plausible to assert that de novo lipogenesis is actually a hallmark of tumor progression [20]. Despite sufficient dietary fat intake, in some tumors, up to 95% of SFA and MUFA are synthesized in cells. This enhanced synthesis leads to changes in lipid tissue profiles, and represents one of the cell reprogramming mechanisms that protect them from lipotoxicity [21]. Given that lung tissue samples are less accessible than blood, the literature on lipid metabolism in cancerous tissues is relatively limited. In our study, the samples were collected from both the malignant and healthy tissues in each patient, allowing the patients to serve as their own control. In this way, genetic consistency was maintained and environmental and lifestyle factors were overcome, thereby more accurately eliminating variations and isolating cancer-specific effects accurately.

As expected, significant differences between cancerous and healthy tissues were noted in both cancer types. No differences were found between male and female patients in the NSCLC group or in the SCLC group, or between smokers and non-smokers. The tumor tissues of both NSCLC and SCLC had higher levels of MUFA (18:1n-7 and total MUFA), n-6 PUFA (AA), and its metabolite 22:4n-6 (DTA). In NSCLC, this rise in MUFA concentration was followed by SFA reduction, which is specifically reflected in the 16:0 decline. This is in line with the literature data indicating higher levels of total MUFA and AA in breast tumor tissue in comparison to normal-appearing tissue [22]. The possible explanation of MUFA prevalence in oncogenic samples is that healthy membranes are rich in SFA, which makes them more rigid and less prone to cellular proliferation. SFA and MUFA exert distinct biological effects, notably influencing cell proliferation, apoptosis, and lipid-induced cytotoxicity, with SFA being particularly noted for their cytotoxic properties [23]. A positive correlation between high levels of tissue MUFA and various cancer types has been reported. They are significantly increased in relation to PUFA in several cancers, including thyroid, esophageal, colorectal, lung, and gastric cancer [24]. These changes in MUFA distribution are possibly a consequence of increased stearoyl-CoA desaturase 1 (SCD1) activity. SCD1, the main isoform of human Δ9 desaturase [25], is a key enzyme driving FA desaturation by converting SFA into MUFA, and it is highly expressed in various tumor tissues, especially in lung cancer cells [26]. It was shown that their inhibition with CVT-11127 reduces lipid synthesis in cancer cells, impairing proliferation through blocking the progression of the cell cycle by triggering apoptosis, with no influence on the proliferation of normal human fibroblasts [27,28]. However, it should be noted that we estimated desaturase and elognase activity as the product-to-precursor ratios of FA, which is common in the literature, and that other factors may have influenced FA concentrations and, consequently, desaturase and elongase activities.

Increased AA is a consequence of the increased Δ6 desaturase activity observed in both analyzed cancerous tissues. This enzyme synthesizes AA from linoleic acid (LA, 18:2n-6), and its upregulated activity in some cancers, such as melanoma, has been reported [29]. The AA itself is a precursor for the synthesis of eicosanoids, biologically active metabolites that have pro-inflammatory effects. Consequently, its elevated concentration is commonly associated with inflammation [30]. In both types of lung cancer, all n-6 PUFA are increased, except LA, which is a precursor of other n-6 PUFA. All of these increased n-6 PUFA are related to inflammation. A slight decrease in anti-inflammatory n-3 PUFA was also noted in NSCLC, while their decrease was more prominent in SCLC, resulting in an elevated n-6/n-3 PUFA ratio. The presence of increased levels of AA was also detected in breast and prostate tumor tissues [22,31], while high levels of their metabolite prostaglandin E2 (PGE2) were measured in numerous malignancies, including prostate, lung, colon, bladder, pancreatic, breast, head, and neck cancer [31]. In our study, a decreased ratio in malignant tissue was observed in patients in the SCLC group. A higher EPA/AA serum ratio was observed in the long-term survivors with SCLC compared to the ratio in short-term survivors [32]. This suggests that lowering AA levels may have positive effects on controlling progression and increasing the chances of recovery. Nevertheless, no changes in AA or other FA were found among different stages in SCLC or NSCLC patients. However, it is important to consider that a small number of patients with early-stage lung cancer may have influenced the lack of statistical significance.

Although NCSLC and SCLC had some similar changes in FA levels, there are also significant differences between the FA metabolism of these two types of lung cancer. For instance, the dihomo-gamma-linoleic acid and DGLA (20:3n-6) percentage was significantly higher in NSCLC tissue, but lower in the SCLC group in comparison to the appropriate control. Increased levels of DGLA were found in the blood of patients with prostate, breast, and gastric carcinoma [12]. DGLA is a substrate for cyclooxygenase (COX) and lipoxygenase (LOX) enzymes. These enzymes could convert DGLA into prostaglandin E1 (PGE1), which exhibits both anti-inflammatory and anti-proliferative properties [33]. PGE1 also inhibits the growth and differentiation of cancer cells, suggesting it could have beneficial effects for NSCLC patients. On the other hand, the decreased concentration of DGLA in SCLC patients could be a consequence of increased Δ5 desaturase activity in this tissue, the role of which is to convert DGLA into AA. This change possibly promotes SCLC cancer progression given that some studies have proved that Δ5 desaturase inhibition prevents the proliferation and migration of tumor cells by leaving DGLA available for oxidation in anticancer metabolites, such as 8-hydroxyoctanoic acid (8-HOA), rather than being utilized for AA synthesis [34]. Additional factors contributing to inflammation are the distribution of n-3 and n-6 FA, as well as the n-6/n-3 ratio. In NSCLC, lower levels of ALA and EPA were documented, while in SCLC, decreased proportions of all individual long-chain n-3 and total n-3 PUFA were found. Reducing the distribution of n-3 PUFA (especially EPA and DHA) can have adverse effects on overall health status, considering the established anti-inflammatory effects of n-3 PUFA [35]. This change has also contributed to a significant increase in the n-6/n-3 ratio in SCLC tissues compared to the healthy control, which was not documented in the NSCLC group. This ratio may be more critical for the development of SCLC, which should be further investigated in cross-sectional studies on a larger number of patients.

Even though it is widely recognized that n-3 PUFA potentially have protective effects, while n-6 PUFA may promote cancerogenesis, some animal and human studies have suggested that their ratio may be more critical than the absolute amount of n-3 PUFA [36]. It was shown that lowering the n-6/n-3 PUFA ratio by supplementation significantly decreased the concentration of serum TNF-α and IL-6 levels in diseased individuals. Simultaneously, the intake of DHA and EPA positively influences the regulation of the blood CRP level [37]. Higher n-6/n-3 proportions detected in malignant tissues of some cancer types tend to confirm the assumption that inflammation is a key step in cancer initiation. Kim et al. established that diets rich in n-6 PUFA induce macrophage infiltration and activate inflammation-related pathways, such as TNF-α, PGE2, NF-κβ, and Wnt signaling, leading to an increased risk of tumorigenesis in colitis-associated carcinogenesis [38].

Through conducting a comparative analysis of PL in NSCLC and SCLC malign tissues, differences in total SFA, PUFA, n-3, and n-6 distribution have been established. In SCLC, a higher level of total SFA (consequence of increased 18:0 and decreased LA concentrations) and a lower level of total PUFA, n-3, and n-6 PL were measured. Previous studies reported that cancer cells with a higher degree of membrane saturation (more MUFA or SFA) are less susceptible to oxidative stress induced by chemotherapeutic agents, while membranes containing higher levels of PUFA are susceptible to peroxidation [39]. Changes in FA composition, such as favoring SFA, protect cancer cells from oxidative stress-induced cell death, thereby interfering with chemotherapy and redox homeostasis [40]. Taking this and the previously described importance of n-3 in tumor development and progression into consideration, it could be assumed that a worse prognosis in SCLC patients could be related to less favorable lipid distribution in the tumor itself. The reprogramming of FA metabolism could be an emerging new therapeutic target.

Although SCD1 is a potential therapeutic target due to the crucial role of MUFA in preventing cell death, no lipid inhibitors have been approved for cancer treatment despite promising preclinical evidence. A significant challenge with cancer anti-metabolic drugs is their disruption of metabolic processes in normal cells, a problem that remains unresolved.

In summary, our results reveal markedly impaired lipid metabolism in lung cancer tissues, with distinct alterations observed between SCLC and NSCLC. Future studies focusing on identifying specific targets and biomarkers through lipidomic and metabolomic profiling are expected to drive the development of personalized treatment strategies for lung cancer. However, the differences in lipid metabolism between SCLC and NSCLC need further investigation to uncover the underlying mechanisms and their potential therapeutic implications. Thus, further research is warranted.

## Figures and Tables

**Table 1:** Lipids and inflammatory parameters in the serum of patients with NSCLC and SCLC.

	NSCLC (*n* = 37)	SCLC (*n* = 27)
TC	4.62 ± 1.06	4.80 ± 1.29
HDL	1.04 ± 0.37	1.01 ± 0.39
LDL	2.97 ± 0.89	3.04 ± 1.30
TG	1.19 ± 0.42	1.65 ± 0.78 **
CRP	39.7 ± 42.6	49.3 ± 54.5
fibrinogen	5.5 ± 1.3	5.1 ± 0.9

NSCLC—non-small cell lung cancer, SCLC—small cell lung cancer, TC—total cholesterol, HDL—high density lipoprotein, LDL—low density lipoprotein, TG—triglycerides, CRP—C-reactive protein; ** *p* ≤ 0.01.

**Table 2:** Fatty acid composition (%) in tumor tissue phospholipids in patients with NSCLC lung cancer and healthy tissue.

Fatty Acids	Control	NSCLC
16:0	30.2 ± 4.9	26.3 ± 4.0 ***
18:0	24.6 ± 5.4	23.6 ± 4.1
SFA	54.8 ± 7.0	49.9 ± 5.9 ***
16:1n-7	1.5 ± 0.9	1.5 ± 1.2
18:1n-7	3.0 ± 1.0	4.4 ± 1.5 ***
18:1n-9	17.4 ± 3.3	18.2 ± 4.2
MUFA	21.9 ± 3.9	24.2 ± 5.3 *
18:2n-6	10.3 ± 3.2	10.2 ± 3.3
18:3n-6	ND	ND
20:3n-6	1.7 ± 0.7	2.0 ± 0.6 *
20:4n-6	7.6 ± 2.4	9.5 ± 2.3 ***
22:4n-6	1.3 ± 0.5	1.9 ± 0.7 ***
n-6 PUFA	21.0 ± 5.2	23.7 ± 5.5 ***
18:3n-3	0.3 ± 0.2	0.2 ± 0.1 **
20:5n-3	0.2 ± 0.1	0.2 ± 0.2
22:5n-3	0.6 ± 0.2	0.6 ± 0.2 **
22:6n-3	1.2 ± 0.8	1.2 ± 0.6
n-3 PUFA	1.9 ± 0.8	2.0 ± 0.8
PUFA	22.9 ± 5.6	25.7 ± 5.8 ***
n-6/n-3 ratio	12.2 ± 3.7	13.0 ± 4.4
EPA/AA	0.03 ± 0.02	0.02 ± 0.02

NSCLC—non-small cell lung carcinoma; SFA—saturated fatty acids; MUFA—monounsaturated fatty acids; PUFA—polyunsaturated fatty acids; ND—not detectable; EPA—Eicosapentaenoic acid; AA—Arachidonic acid; * *p* < 0.05, ** *p* < 0.01, *** *p* < 0.001; (*n* = 37).

**Table 3:** The estimated desaturase and elongase activities in patients with NSCLC; tumor and control tissues.

Desaturase and Elongase	Control	NSCLC
Δ5	5.1 ± 2.4	5.1 ± 1.6
Δ6	0.17 ± 0.05	0.20 ± 0.04 ***
Δ9	0.7 ± 0.2	0.8 ± 0.3
SCD1	0.05 ± 0.03	0.05 ± 0.05
SCD2	0.75 ± 0.25	0.81 ± 0.33
Elongase	0.8 ± 0.2	0.9 ± 0.2

NSCLC—non-small cell lung carcinoma, SCD—stearoyl-CoA desaturase; *** *p* < 0.001; (*n* = 37).

**Table 4:** Fatty acid composition (%) in tumor tissue phospholipids in patients with SCLC lung cancer and healthy tissue.

Fatty Acids	Control	SCLC
16:0	28.8 ± 4.5	28.0 ± 2.7
18:0	28.4 ± 6.7	27.4 ± 5.1
SFA	57.2 ± 7.8	55.4 ± 6.6
16:1n-7	1.3 ± 1.0	1.1 ± 0.7
18:1n-7	3.0 ± 0.7	4.4 ± 1.2 ***
18:1n-9	16.1 ± 3.1	18.4 ± 4.0 *
MUFA	20.5 ± 3.4	23.9 ± 4.4 **
18:2n-6	10.3 ± 3.5	6.6 ± 2.7 ***
18:3n-6	ND	ND
20:3n-6	1.9 ± 0.7	1.5 ± 0.5 *
20:4n-6	6.7 ± 2.2	9.0 ± 2.9 ***
22:4n-6	1.2 ± 0.5	1.9 ± 1.0 ***
n-6 PUFA	20.1 ± 5.8	19.1 ± 4.9
18:3n-3	0.2 ± 0.2	0.2 ± 0.1
20:5n-3	0.2 ± 0.1	0.1 ± 0.1
22:5n-3	0.6 ± 0.4	0.4 ± 0.3 ***
22:6n-3	1.1 ± 0.4	0.9 ± 0.3 ***
n-3 PUFA	1.9 ± 0.6	1.4 ± 0.6 ***
PUFA	22.1 ± 6.1	20.5 ± 5.1 †
n-6/n-3 ratio	11.0 ± 3.6	15.4 ± 4.4 ***
EPA/AA	0.03 ± 0.03	0.02 ± 0.02 *

SCLC—small cell lung carcinoma; SFA—saturated fatty acids; MUFA—monounsaturated fatty acids; PUFA—polyunsaturated fatty acids; ND—not detectable; EPA—Eicosapentaenoic acid; AA—Arachidonic acid; * *p* < 0.05, ** *p* < 0.01, *** *p* < 0.001; † *p* = 0.51; (*n* = 27).

**Table 5:** The estimated desaturase and elongase activities in patients with SCLC; tumor and control tissues.

Desaturase and Elongase	Control	SCLC
Δ5	4.0 ± 1.6	6.1 ± 2.4 **
Δ6	0.18 ± 0.05	0.25 ± 0.07 ***
Δ9	0.6 ± 0.2	0.7 ± 0.3 *
SCD1	0.04 ± 0.02	0.04 ± 0.02 †
SCD2	0.64 ± 0.19	0.73 ± 0.30
Elongase	1.0 ± 0.3	1.0 ± 0.2

SCLC—non-small cell lung carcinoma, SCD—stearoyl-CoA desaturase; * *p* < 0.05, ** *p* < 0.01, *** *p* < 0.001; (*n* = 27); † *p* = 0.07.

## Data Availability

Data are available upon reasonable request to the corresponding author.
